# Synthesis and Evaluation of Novel Organogermanium Sesquioxides As Antitumor Agents

**DOI:** 10.1155/2009/908625

**Published:** 2009-06-02

**Authors:** Chun Li Zhang, Tai Hua Li, Shuang Huan Niu, Rong Fu Wang, Zhan Li Fu, Feng Qin Guo, Ming Yang

**Affiliations:** ^1^State Key Laboratory of Natural and Biomimetic Drugs, Peking University Health Science Center, Beijing 100083, China; ^2^Department of Nuclear Medicine, Peking University First Hospital, Beijing 100034, China; ^3^Department of Chemistry, Beijing Normal University, Beijing 100875, China

## Abstract

Five new organogermanium sesquioxides have been synthesized and characterized by elemental analysis and IR spectra. All the compounds were tested for antitumor activities against KB, HCT, and Bel cells in vitro. Compound 5 (*γ*-thiocarbamido propyl germanium sesquioxide) showed excellent antitumor activity, and its inhibition yield to KB, HCT, and Bel cells was 92.9%, 84.9%, and 70.9%, respectively. A rapid method was described for the labeling compound 5 with ^99m^Tc, and the optimum labeling conditions were investigated. The labeling yield is above 90% in pH 7.0, 20°C, reaction time greater than 10 minutes, 1 mg of compound 5, and 0.075∼0.1 mg of SnCl_2_. The biodistribution of ^99m^Tc labeled compound 5 in nude mice bearing human colonic xenografts was studied. The result showed that the tumor uptakes were 0.73, 0.97, 0.87, and 0.62 ID%/g at 1-hour, 3-hour, 6-hour, and 20-hour postinjection, respectively. T/NT (the uptake ratio for per gram of tumor over normal tissues) was 18.3 for tumor versus brain and 5.81 for tumor versus muscle at 20-hour postinjection. The tumor clearance was slow. The results showed that compound 5 may be developed to be a suitable cancer therapeutic agent.

## 1. Introduction

Germanium is a constituent of many medicine plants such as ginseng root, and it is considered to play an important role in the pharmacological effects of the plants [1]. It is reported that many organogermanium compounds can inhibit tumor and metastatic growth and modify immune response by inducing interferon-*γ* (IFN-*γ*), enhancing NK cell activity, and increasing peritoneal macrophage activity [2–5]. Besides, they are of extremely low toxicity [5, 6]. So, many researches focus on searching for effective and low-toxic antitumor drugs from organogermanium compounds [ 7–13]. Several types of organogermanium compounds have been investigated and found to possess antitumor effect [14–16], such as tetraalkylgermanium and alkylgermanium halide [17], spirogermanium [18], germanium sesquisulfide [19] or sesquioxide [20, 21], germatranes [22], Ge-porphyrinates, and germanium compounds containing phosphorus [23–25], of which Ge-132 (carboxyethylgermanium sesquioxide) has been clinically used [26, 27].

For developing more effective antitumor agents from organogermanium sesquioxides, we synthesized five new organogermanium sesquioxides and investigated their antitumor activities against KB, HCT, and Bel cells. The compound with the highest antitumor activity was selected for ^99m^Tc labeling and biodistribution study in nude mice bearing human colonic xenografts.

## 2. Experimental

### 2.1. Agents, Experimental Animals, and Instrumentation

All commercially available chemicals were of analytical grade and used without further purification. ^99m^TcO_4_^−^ was provided by Syncor company with radiochemical purity greater than 98%.

Experimental animals (Balb/c nude mice) were provided by Experimental Animal Department of Chinese Academy of Medical Sciences. The nude mice were implanted with human colonic carcinoma cells (LoVo cells). When the tumor size was about 1 cm, the mice were used for the experiment.

IR spectra were recorded on a Bruker Equinox 55 spectrometer, US. Elemental analyses were determined on a Yanaco CHN Corder MT-3 elemental analyzer. Mass spectrum (MS) was detected on a Bruker APEX IV FTMS mass spectrometer. Radioactivity was counted on a HY-901 *γ* counter provided by Sixin company.

### 2.2. Chemical Synthesis

**Figure 1:**
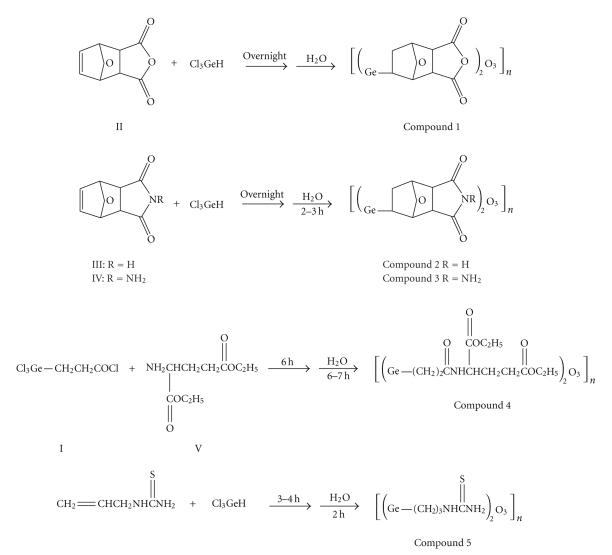
Scheme of synthesis route.

The scheme of synthesis route is shown in Figure 1. The details of synthetic method were described by Niu [28].

#### 2.2.1. Reagents

**(1) Cl_3_GeH**

Cl_3_GeH was prepared by the method reported by Bai et al. [29]. 0.01 mol of GeO_2_, 2 mL of 50% H_3_PO_2_, and 9 mL of HCl solution (11.9 M) were mixed and refluxed for 4 hours. A colorless Cl_3_GeH solution was obtained. The solution was extracted three times with ether to give Cl_3_GeH solution in ether.

**(2) *β*-Chloroformyl Ethyl Germanium Trichloride (I)**

*β*-chloroformyl ethyl germanium trichloride (I) was prepared by the method reported by Bai et al. [29]. 0.01 mol propenoic acid was added dropwise to 0.01 mol Cl_3_GeH in HCl solution. A sticky precipitate was formed. After stirring for 4 hours, the precipitate was filtered and recrystallized with CH_2_Cl_2_ to obtain 1.5 g (0.0055 mol) of I. 60% yield; mp: 70~72°C.

**(3) 7-Oxabicyclo [2.2.1] Hept-5-Ene-2, 3-Dicarboxylic Acid Anhydride (II)**

7-oxabicyclo [2.2.1] hept-5-ene-2, 3-dicarboxylic acid anhydride (II) was prepared by the method reported by Tian et al. [30]. 0.5 mol of maleic anhydride, 0.6 mol of furan, and 50 mL of anhydrous tetrahydrofuran were mixed and stirred to dissolve the maleic anhydride at 37°C. The solution was placed in room temperature for 2~3 days until the crystals were formed. The crystals were filtered, dried, and then recrystallized with ethanol to obtain II. mp: 125~127°C.

**(4) 7-Oxabicyclo [2.2.1] Hept-5-Ene-2,3-Dicarboximine (III)**

7-oxabicyclo [2.2.1] hept-5-ene-2,3-dicarboximine (III) was prepared by the method reported by Liu et al. [31]. II was added to 25 mL of ammonia liquor (14.8 M) under stirring. The mixture was refluxed in boiling water for 1 hour to yield yellow solution. The solution was cooled and stood overnight. Needle crystals were formed. The crystals were filtered, dried, and then recrystallized with 95% ethanol to obtain III. mp: 173~175°C.

**(5) N-Amino-7-Oxabicyclo [2.2.1] Hept-5-Ene-2,3-Dicarboximine (IV)**

N-amino-7-oxabicyclo [2.2.1] hept-5-ene-2,3-dicarboximine (IV) was prepared by the method reported by Liu et al. [31]. 5 mL of 50% hydrated hydrazine was added dropwise to 0.06 mol of I in water. The mixture was refluxed in boiling water for 1 hour to yield purple solution and then stood overnight. Lamellar crystals were formed. The crystals were filtered, dried, and then recrystallized with distilled water to obtain IV. mp: 145~146°C.

**(6) Ethyl Glutamate (V)**

To prepare ethyl glutamate (V), a mixture of glutamic acid (0.2 mol) and anhydrous ethanol (100 mL) was refluxed in an oil bath. Dry hydrochloride gas was ventilated until the amino acid was dissolved completely. Then we stopped heating and continued to stir the reaction mixture for 1 hour. Evaporation of the excess ethanol in vacuo resulted in light yellow oil. 40 mL of distilled water was added, and the pH of the solution was increased by the addition of 8 g of NaOH and stirred for 2 hours. The reaction mixture was extracted three times with ethyl acetate (15 mL), dried (with anhydrous Na_2_SO_4_), and concentrated in vacuo to give V as a light yellow oil.

#### 2.2.2. Synthesis of Germanium Sesquioxides of 7-Oxabicyclo [2.2.1] Hept-2,3-Dicarboxylic Acid Anhydride, and 7-Oxabicyclo [2.2.1] Hept-2,3-Dicarboximine Derivatives (Compound 1, 2, 3)

To a solution of II or III, IV (0.01 mol) in an anhydrous solvent was added dropwise Cl_3_GeH (0.01 mol), and the solution was stirred overnight. The solvent was evaporated in vacuo, and the residue was dissolved in distilled water (20 mL) and stirred for 2~3 hours to give a hydrolyzed product as a precipitate. The precipitate was filtered, washed with aforementioned solvent and distilled water, and dried to afford compound 1 or 2, 3.

Compound 1:

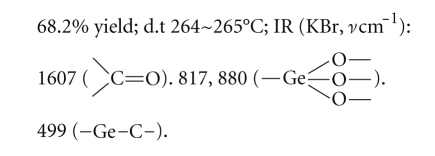

Elemental analysis: C: 36.21%, H: 2.62% (found); C: 36.42%, H: 2.66% (calcd.).

Compound 2:

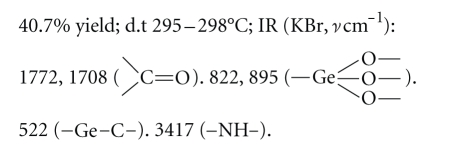

Elemental analysis: C: 36.38%, H: 3.00%, N: 5.29% (found); C: 36.56%, H: 3.05%, N: 5.33% (calcd.).

Compound 3:

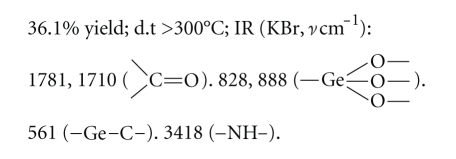

Elemental analysis: C: 34.63%, H: 3.29%, N: 10.01% (found); C: 34.58%, H: 3.24%, N: 10.09% (calcd.).

Compound 4:

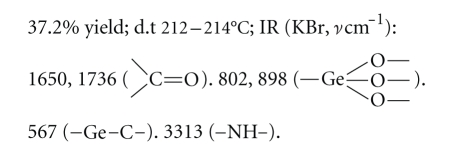

Elemental analysis: C: 41.02%, H: 5.64%, N: 4.01% (found); C: 40.61%, H: 5.64%, N: 3.95% (calcd.).

Compound 5:

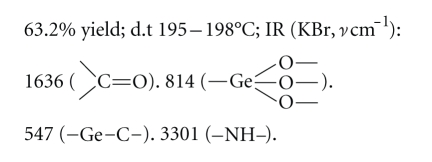

Elemental analysis: C: 21.96%, H: 4.23%, N: 13.09% (found); C: 22.47%, H: 4.21%, N: 13.11% (calcd.).

#### 2.2.3. Synthesis of Germanium Sesquioxide of Ethyl Glutamate (Compound 4)

To a vigorously stirred solution of I in ether (10 mL) was added dropwise a solution of V (4.0 g) in ether, and the solution was stirred for 6 hours. A white sticky precipitate was formed and filtered off. The filtrate was evaporated in vacuo to remove ether. Adding 10 mL of distilled water and stirring for 6~7 hours yielded a white precipitate. The precipitate was filtered, washed three times with distilled water, and dried to give compound 4 as a white or light yellow powder.

#### 2.2.4. Synthesis of *γ*-Thiocarbamido Propyl Germanium Sesquioxide (Compound 5)

To a solution of propenyl thiocarbamide (0.01 mol) in anhydrous ethynol was added dropwise a solution of Cl_3_GeH (0.01 mol) in ether, and the solution was stirred for 3~4 hours. The solvent was evaporated in vacuo to give an oily residue. Addition of distilled water (20 mL) yielded a bright yellow precipitate. The solution was kept stirring for 2 hours. The precipitate was filtered, washed three times with distilled water, and dried to give compound 5.

### 2.3. Antitumor Activity

The antitumor activity in vitro was assayed by the MTT method [32] using DMSO as solvent. The tumor inhibition yield and IC_50_ of the five organogermanium sesquioxides and Ge-132 against KB (human nasopharyngeal cancer), HCT (human colonic cancer), and Bel (human liver cancer) cells were measured.

### 2.4. Radiolabeling with ^99m^Tc

Since compound 5 showed best antitumor activity, it was selected for further radiolabeling and biodistribution studies. To the solution of compound 5 in NaOH solution (2 mol/L), 100 *μ*L freshly prepared SnCl_2_ *·*H_2_O solution was added, and pH was adjusted to the required value. 3700~5500 kBq of ^99m^TcO_4_^−^ eluate was added, and the solution was stirred for 5~25 minutes to give the labeled compound. Labeling yield was measured by TLC with polyamide membrane in the solvent of (CH_3_)_2_CHOH : NH_4_OH : H_2_O = 15 : 13 : 5 (v/v/v). Rf: labeled compound: 0.3~0.4, ^99m^TcO_4_^−^: 0.5~0.6, ^99m^TcO*·* *x*H_2_O: 0~0.1. Reaction conditions were optimized by varying the concentration of germanium sesquioxide solution and reducing agent (SnCl_2_), pH, temperature, and reaction time.

### 2.5. Biodistribution

Twelve nude mice bearing human colonic cancer xenografts were injected in tail vein with 555~740 kBq of ^99m^Tc labeled compound 5. Three animals were sacrified at 1-hour, 3-hour, 6-hour, and 20-hour postinjection, respectively. Tissues and organs were excised, rinsed, weighted, and counted in a *γ* counter. The uptake percentage of the injected dose per gram of the measured organ (ID %/g) and the ratio of ID %/g of tumor over normal tissues (T/NT) were calculated.

## 3. Results

### 3.1. Preparations

The compounds were prepared under mild condition. All the five compounds are powder and stable under ordinary conditions. They all are not soluble in water, alcohol, slightly soluble in DMSO, but compounds 3, 4, and 5 are easily soluble in 2 mol/L of NaOH solution.

The elemental analysis data and IR spectra were consistent with the expected structure of the compounds. In IR spectra, the wide absorption band at 800~900 cm^−1^ corresponds to the characteristic region of *ν*Ge–O. The weak absorption peak at about 550 cm^−1^ corresponds to the characteristic region of *ν*Ge–C.

MS was performed on compound 5 dissolved in the mixture of DMSO and methanol. The solvent was selected because DMSO was the solvent for measuring antitumor activity, and methanol was used to decrease the viscosity of the solution to facilitate MS detection. MS showed the compound containing two germanium atoms with *m*/*z* 456.815. The *m*/*z* value corresponds to the molecular weight of the formula [Ge(CH_2_)_3_NHCSNHCH_3_]_2_O_3_ which may be formed by methylation of [Ge(CH_2_)_3_NHCSNH_2_]_2_O_3_ by methanol in DMSO.

### 3.2. Antitumor Activity

The antitumor activities of the five compounds and Ge-132 at the concentration of 1, 10, and 50 *μ*g/mL were tested. All the five compounds showed no antitumor activity at the concentration of 1 and 10 *μ*g/mL. The antitumor activities of the compounds at the concentration of 50 *μ*g/mL are listed in Table 1. The results of bioassay show that compounds 4, 5 exhibit certain activities against cancer cells, but compounds 1, 2, 3 show no antitumor activity. Compound 5 shows the highest antitumor activity. Its inhibition yields to KB, HCT, and Bel cells are 92.9%, 84.9%, and 70.9%, respectively. IC_50_ is smaller than 10^−7^ M (IC_50_ < 10^−7^ M is considered as strong inhibition, IC_50_ = 10^−7^~10^−6^ M as medium inhibition, and IC_50_ = 10^−6^~10^−5^ M as weak inhibition). Ge-132 shows antitumor activity in vitro only to HCT cell, and the inhibition yield is 31.4%.

### 3.3. ^99m^Tc Labeling

**Figure 2:**
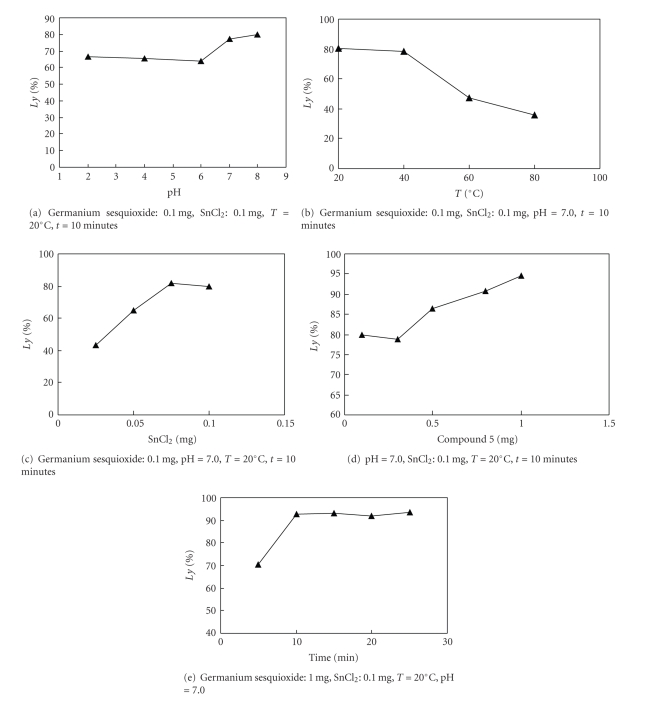
Labeling yields (*Ly*s) at different conditions.

Labeling yields of compound 5 at different conditions are showed in Figure 2. The compound shows maximum labeling yield of above 90% at 1 mg of the germanium sesquioxide, pH 7.0 ∼ 8.0, 20°C, with 0.075~0.1 mg of SnCl_2_ as reducing agent for more than 10 minutes of reaction.

### 3.4. Biodistribution

The result of the biodistribution study of compound 5 in nude mice is tabulated in Table 2. The uptakes of the compound in heart, brain, and muscle are low. It shows high uptake in liver, kidney, and lung. The lung has the highest uptake at 3-hour and 6-hour postinjection.

**Figure 3:**
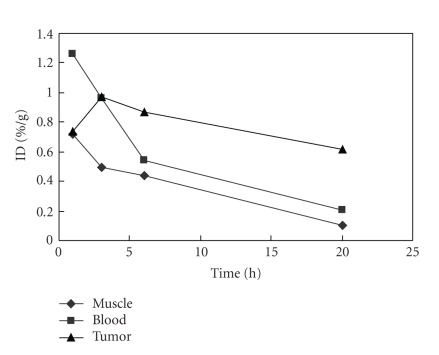
ID %/g of compound 5 in tumor, muscle, and blood at different time points.

Tumor uptakes of compound 5 are 0.73, 0.97, 0.87, and 0.62 ID %/g at 1-hour, 3-hour, 6-hour, and 20-hour postinjection, respectively, which are higher than those of muscle, heart, and brain but lower than those of kidney, liver, and lung. The clearance of the compound in tumor is relatively low (Figure 3). At 20-hour postinjection, the tumor uptake is higher than that of all other tissues except kidney, liver, and lung. The maximum T/NT is the ratio of tumor to brain (18.30) and followed by the ratio of tumor to muscle (5.81) at 20-hour postinjection. T/NT for tumor versus blood increases with time with the maximum of 3.01 at 20-hour postinjection (Table 3). The result of its relatively high tumor uptake and low tumor clearance as well as its high tumor inhibition yield suggests that compound 5 may be developed to be a suitable agent for cancer therapy.

## 4. Discussion

Mironov et al. synthesized bis (2-carboxyethylgermanium) sesquioxide (now known as Ge-132) in the 1960s [33]. In the 1970s, Japanese scholar K. Asai. synthesized organogermanium pharmaceuticals with wide spectra of pharmacological activities, in which he found Ge-132 showing antitumor activity [34]. Since then, a lot of studies showed that many organogermanium compounds can modify immune function, inhibit tumor cell proliferation [35], induce tumor cell apoptosis [36], and they are efficient in treating and preventing cancers [37–40]. Besides, they can also relieve cancer-induced pain [41], but the main problem of organogermanium compounds used as antitumor agents is their low pharmacological effects, which makes them can only be used as auxiliary agents in tumor therapy. So, development of more effective antitumor organogermanium compounds is a valuable subject.

In this study, five new organogermanium compounds were synthesized and characterized by elemental analysis data and IR spectra. The elemental analysis data and IR spectra were consistent with the expected structure of the compounds. In IR spectra, the wide absorption band at 800~900 cm^−1^ of the characteristic region of *ν*Ge–O provides support for Ge–O network structure. The weak absorption peak at about 550 cm^−1^ corresponds to the characteristic region of *ν*Ge–C. The result shows that Ge–O network structure and Ge–C bond exist in the compounds.

One of the characteristics of germanium compounds is polymorphism [5]. The basic formula of germanium sesquioxides is (GeR)_2_O_3_. So germanium sesquioxides can be represented by [(GeR)_2_O_3_]_n_ where R is the group conjugated with germanium atom and *n* is the degree of polymerization. The degree of polymerization strongly influences the toxicity of the compounds. It is reported that the value of “*n*” depends on the way in which a solid product separates out of a solution of the compound. Particle size and bulk density of the dried solid in batch-to-batch comparisons are notoriously variable. Although there may be many polymorphic solid forms, the aqueous solutions, once they are formed, are identical [5]. In this study, the “*n*” value of compound 5 in the mixture of DMSO and methanol was detected. The result showed that the compound existed in dimer form in the solution.

Two of the five compounds showed certain antitumor activity at the concentration of 50 *μ*g/mL. Compound 5 showed very high antitumor activity with the inhibition yields of 92.9%, 84.9%, and 70.9% to KB, HCT, and Bel cells at the concentration of 50 *μ*g/mL (0.117 mmol/L), respectively. Compound 4 showed antitumor activity only to HCT cell with the inhibition yield of 41.5%. The antitumor activities of compound 4 and compound 5 were higher than that of Ge-132 which has been clinically used. But Ge-132 showed its in vivo antitumor effect partly due to its inducing the antitumor immunity of the host. Whether the new organogermanium compounds synthesized have the in vivo immunopotentiating activity needs to be further investigated.

Since many organogermanium compounds have radiosensitive effect [36], it is expected that radiolabeled organogermanium compounds may be more effective in the therapy of cancer by synergic effect of cytotoxicity of the compounds and radiation effect. We investigated the method of labeling compound 5 with ^99m^Tc. The result showed that the compound can be successfully labeled with labeling efficiency greater than 90%. The therapeutic radionuclide, ^186^Re or ^188^Re, has similar chemical property to ^99m^Tc. So it is hopeful to label the organogermanium compound with ^186^Re or ^188^Re for the tumor therapy study. This work will be done later.

Labeling compound 5 with ^99m^Tc also allowed the biodistribution study of the compound in nude mice bearing human colonic cancer. The biodistribution data showed that the compound was mainly concentrated in kidney, lung, and next in liver. Tumor uptake was higher than that of muscle, heart, and brain, but lower than that of kidney, liver, and lung after 3-hour postinjection. The clearance of the compound in tumor was relatively low. The result showed that the compound has relative high tumor uptake and low tumor clearance, which facilitates using the compound in cancer therapy.

## 5. Conclusion

A new class of organogermanium sesquioxides was synthesized and characterized. The antitumor activity, ^99m^Tc labeling, and biodistribution were studied. Of the five compounds, compound 5 shows excellent antitumor activity, high tumor uptake, and slow clearance in tumor. It may be developed to be a suitable cancer therapeutic agent. The in vivo antitumor effects of the organogermanium sesquioxides need to be further investigated.

## Figures and Tables

**Table 1:** Inhibition yields (%) of the five compounds and Ge-132 at the concentration of 50 *μ*g/mL.

Cells	Compound 1	Compound 2	Compound 3	Compound 4	Compound 5	Ge-132
KB	0	0	0	0	92.9	0
HCT	0	0	0	41.5	84.9	31.4
Bel	0	0	0	0	70.9	0

**Table 2:** Biodistribution of ^99m^Tc labeled compound 5 in nude mice bearing human colonic cancer xenografts at various time intervals (% ID/g, *n* = 3, $\overset{¯}{x}\pm \text{SD}$).

Tissues	1 h	3 h	6 h	20 h
Stomach	10.10 ± 3.89	5.57 ± 0.89	1.27 ± 0.25	0.59 ± 0.18
Small intestine	1.29 ± 0.58	1.06 ± 0.11	3.09 ± 3.27	0.33 ± 0.14
Kidney	8.86 ± 3.23	7.07 ± 0.50	6.65 ± 0.41	5.89 ± 0.96
Liver	2.96 ± 0.99	3.65 ± 0.44	2.59 ± 0.07	1.99 ± 0.74
Lung	4.66 ± 0.24	8.24 ± 2.24	12.40 ± 4.94	4.56 ± 3.28
Muscle	0.72 ± 0.84	0.49 ± 0.34	0.44 ± 0.34	0.11 ± 0.04
Heart	0.64 ± 0.23	0.58 ± 0.09	0.34 ± 0.00	0.21 ± 0.05
Brain	0.07 ± 0.02	0.11 ± 0.06	0.07 ± 0.01	0.03 ± 0.01
Blood	1.26 ± 0.28	0.97 ± 0.18	0.55 ± 0.06	0.21 ± 0.06
Tumor	0.73 ± 0.10	0.97 ± 0.20	0.87 ± 0.04	0.62 ± 0.23

**Table 3:** T/NT of ^99m^Tc labeled compound 5 at various time intervals in nude mice bearing human colonic cancer xenografts (*n* = 3, $\overset{¯}{x}\pm \text{SD}$).

Tissues	1 h	3 h	6 h	20 h
Stomach	0.07 ± 0.02	0.18 ± 0.06	0.69 ± 0.10	1.02 ± 0.07
Small intestine	0.63 ± 0.20	0.92 ± 0.15	0.62 ± 0.64	1.93 ± 0.47
Kidney	0.09 ± 0.04	0.14 ± 0.02	0.13 ± 0.02	0.10 ± 0.02
Liver	0.26 ± 0.06	0.27 ± 0.08	0.34 ± 0.03	0.31 ± 0.02
Lung	0.16 ± 0.02	0.12 ± 0.01	0.04 ± 0.01	0.27 ± 0.28
Muscle	1.01 ± 1.18	3.88 ± 4.19	2.77 ± 2.05	5.81 ± 0.93
Heart	1.22 ± 0.34	1.66 ± 0.19	2.54 ± 0.15	3.00 ± 1.05
Brain	10.70 ± 3.34	9.99 ± 4.53	12.90 ± 2.95	18.30 ± 5.72
Blood	0.72 ± 0.23	1.18 ± 0.02	1.60 ± 0.26	3.01 ± 0.98
